# Molecular assessment of *kelch13* non-synonymous mutations in *Plasmodium falciparum* isolates from Central African Republic (2017–2019)

**DOI:** 10.1186/s12936-020-03264-y

**Published:** 2020-05-24

**Authors:** Romaric Nzoumbou-Boko, Chris-Boris Gildas Panté-Wockama, Carine Ngoagoni, Nathalie Petiot, Eric Legrand, Ulrich Vickos, Jean-Chrysostome Gody, Alexandre Manirakiza, Christophe Ndoua, Jean-Pierre Lombart, Didier Ménard

**Affiliations:** 1 Laboratoire de Parasitologie, Institut Pasteur de Bangui, BP 923, Bangui, Central African Republic; 2 Laboratoire de Biochimie, Université de Bangui, BP 1450, Bangui, Central African Republic; 3 Service d’Entomologie Médicale, Institut Pasteur de Bangui, BP 923, Bangui, Central African Republic; 4 Unité Génétique du Paludisme et Résistance, Département de Parasites et Insectes Vecteurs, Institut Pasteur, 25-28 Rue du Dr Roux, 75015 Paris, France; 5 Complexe Pédiatrique de Bangui, Bangui, Central African Republic; 6 Unité d’Épidémiologie, Institut Pasteur de Bangui, BP 923, Bangui, Central African Republic; 7 Programme National de Lutte contre le Paludisme, Ministère de la Santé Publique, Bangui, Central African Republic

**Keywords:** Malaria, *Plasmodium falciparum*, Antimalarial drug resistance, Artemisinin, *Pfkelch13*, Bangui, Central African Republic

## Abstract

**Background:**

Over the last decade, artemisinin-based combination therapy (ACT) has contributed substantially to the decrease in malaria-related morbidity and mortality. The emergence of *Plasmodium falciparum* parasites resistant to artemisinin derivatives in Southeast Asia and the risk of their spread or of local emergence in sub-Saharan Africa are a major threat to public health. This study thus set out to estimate the proportion of *P. falciparum* isolates, with *Pfkelch13* gene mutations associated with artemisinin resistance previously detected in Southeast Asia.

**Methods:**

Blood samples were collected in two sites of Bangui, the capital of the Central African Republic (CAR) from 2017 to 2019. DNA was extracted and nested PCR were carried out to detect *Plasmodium* species and mutations in the propeller domain of the *Pfkelch13* gene for *P. falciparum* samples.

**Results:**

A total of 255 *P. falciparum* samples were analysed. *Plasmodium ovale* DNA was found in four samples (1.57%, 4/255). Among the 187 samples with interpretable *Pfkelch13* sequences, four samples presented a mutation (2.1%, 4/187), including one non-synonymous mutation (Y653N) (0.5%, 1/187). This mutation has never been described as associated with artemisinin resistance in Southeast Asia and its in vitro phenotype is unknown.

**Conclusion:**

This preliminary study indicates the absence of *Pfkelch13* mutant associated with artemisinin resistance in Bangui. However, this limited study needs to be extended by collecting samples across the whole country along with the evaluation of in vitro and in vivo phenotype profiles of *Pfkelch13* mutant parasites to estimate the risk of artemisinin resistance in the CAR.

## Background

The introduction of artemisinin-based combination therapy (ACT) as the first-line treatment for *Plasmodium falciparum* malaria worldwide has greatly helped to reduce the morbidity and the mortality due to malaria from 2010 (251 million cases and 585,000 deaths) to 2017 (228 million cases and 405,000 deaths) [1, 2]. The global implementation of ACT has thus inspired hope, but soon became an issue of concern due to the emergence of artemisinin-resistant parasites in 2006–2007 in the Greater Mekong subregion, and the risk of spreading of these resistant strains to sub-Saharan Africa where malaria transmission and burden are high [3, 4]. Since then, many sub-Saharan African countries have enhanced their surveillance system to assess the efficacy of ACT in clinical drug efficacy studies and to detect artemisinin resistant parasites by in vitro susceptibility testing or *Pfkelch13* genotyping [5, 6].

In the Central African Republic (CAR), ACT has been used since 2006 as first- and second-line treatments [7]. Set up in October 2016 and based on the WHO Global Technical Strategy for Malaria 2016–2030, the national CAR policies for malaria control emphasize surveillance of clinical efficacy, in vitro susceptibility testing and screening for molecular markers associated with anti-malarial drug resistance. Nonetheless, there are very few reliable data available. Two studies on in vitro sensitivity tests conducted in Bangui in 2004 (before ACT was introduced) and in 2014 showed that 100% of the circulating *P. falciparum* strains were sensitive to the main artemisinin-based drug combinations [8, 9]. A clinical study assessing the efficacy of ACT was conducted in Bangui in 2010 and showed a high level of clinical and parasitological response rate estimated to 100% for the artemether–lumefantrine (AL) and artesunate–amodiaquine (ASAQ) [10]. The sole study assessing the prevalence of mutant *Pfkelch13* parasites was conducted in 2014 in Bangui and showed a frequency of 4.5% non-synonymous mutations (Q468R, W470Stop, K480R, L505S, Y519C, S522C, N537D, G545E, I552M, W565Stop, V566I, S577P, A578S, F583L, V589I, G591D, E606G, E612G, Q633R, I640V, D641G), all not validated to confer artemisinin resistance [11].

In addition to the lack of data on artemisinin resistance, since 2012, the CAR has been experiencing unprecedented social unrest and political instability, leading to the arrival of thousands of expatriate civilians and foreign troops, some of whom come from countries located in known multidrug-resistant malaria areas. The presence in the CAR of troops from Cambodia and Thailand, two countries located in the area where artemisinin-resistance have emerged, and from Bangladesh, Bhutan and Nepal, countries that neighbour this epicentre of emergence, may lead to the potential spread of multidrug-resistant *P. falciparum* in Africa in general, and to the CAR in particular.

This concern is further heightened by studies (Ménard, pers. commun.) that have shown a proportion of 0.9% asymptomatic carriers in the Cambodian military personnel sent to Africa for peacekeeping missions (18 *P. falciparum* infections among the 1950 military personnel tested between June 2014 and June 2017). Moreover, there are a multiplicity of factors that may favour the emergence, the introduction and—more importantly, the selection—of drug-resistant malaria strains, particularly those resistant to artemisinin derivatives. These factors include population migration within the CAR or emigration to neighbouring countries, the deplorable living conditions in displaced persons and refugee camps, the illicit trafficking of fake anti-malarial drugs in local markets. Since 2014, several single non-synonymous mutations in the propeller domain of the *Pfkelch13* gene have been associated with resistance to artemisinin derivatives, defined clinically by a delayed clearance of *P. falciparum* during the three-day course of ACT or by the increase in the number of parasites (ring stages) resistant to a pulse of 700 nM of DHA as expressed in the ring-stage survival assay (RSA) [12]. Since then, in vivo clinical drug efficacy study and in vitro parasite susceptibility testing along with screening for specific mutations in the *Pfkelch13* gene are the recommended approach for the surveillance of the efficacy of ACT [13]. However, in vivo and in vitro approaches have some practical issues regarding their elaborate protocols and the follow-up of patients for more than 1 month post-infection. Therefore, screening for mutations to detect potential resistance markers may be a useful, efficient alternative. This study thus set out to investigate *kelch13* polymorphism in *P. falciparum* isolates collected in Bangui, the capital of the CAR.

## Methods

### Study site and period

The study was carried out in Bangui, where malaria transmission is holoendemic, on samples taken from symptomatic malaria patients at two health centres during two different periods: (1) at the Institut Pasteur in Bangui (IPB) between September 2017 and February 2018 and (2) at the Bangui Paediatric Complex (BPC) between November 2018 and March 2019. Both centres are located in the 1st district of Bangui, but patients come from all districts.

### Study population and sampling

The study population was made up of patients visiting the IPB and the BPC for malarial symptoms. Patients included in the study showed positive Giemsa-stained thick blood smears, and blood samples and demographic data were available for them. All blood samples were collected in EDTA blood collection tubes. Some of the sampled blood was then spotted on filter paper for further analyses.

### *Plasmodium* species identification

DNA was extracted using the Chelex-100 method on the dried blood spot samples [14]. Extracted DNA was used to identify *Plasmodium* species using the technique described by Singh et al. [15]. The targeted 18S (SSU) rRNA gene common to all four *Plasmodium* species was amplified using a specific primer pair (PCR1) and then species-specific primers were used to screen for each individual *Plasmodium* species (PCR2) (Table 1). After migration, PCR products were observed under UV light and the bands were compared with the positive controls for species identification.

**Table 1:** Primers sequences used to identify *Plasmodium* species and size of PCR products

	PCR	Primers names	Primers sequences	Size of PCR products
*Plasmodium* genus	PCR1	rPLU5 rPLU6	5′-cctgttgttgccttaaacttc-3′ 5′-ttaaaattgttgcagttaaaacg-3′	–
*P. falciparum*	Nested	rFAL-F rFAL-R	5′-cttttgagaggttttgttactttgagtaa-3′ 5′-tattccatgctgtagtattcaaacaaaa-3′	205 bp
*P. ovale*		rOVA-F rOVA-R	5′-ttttgaagaatacattaggatacaattaatg-3′ 5′-catcgttcctctaagaagctttaccct-3′	800 bp
*P. vivax*		rVIV-F rVIV-R	5′-acgcttctagcttaatccacataact-3′ 5′-atttactcaaagtaacaaggacttccaagc-3′	120 pb
*P. malariae*		rMAL-F rMAL-R	5′-ataacatagttgtacgttaagaataaccgc-3′ 5′-aaaattcccatgcataaaaaattatacaaa-3′	144 bp
*Pfkelch13*	PCR1	K13_PCR_F K13_PCR_R	5′-cggagtgaccaaatctggga-3′ 5′-gggaatctggtggtaacagc-3′	–
	Nested	K13_N1_F K13_N1_R	5′-gccaagctgccattcatttg-3′ 5′-gccttgttgaaagaagcaga-3′	849 pb

### Detection of mutations in the *Pfkelch13* gene

A portion of the *Pfkelch13* gene was amplified from the DNA extract on confirmed *P. falciparum* samples using the method described by Ariey et al. [12]. Briefly, amplification of the *Pfkelch13*-propeller domain (codons 440–680, 720 bp) was performed as following. Five μl DNA was amplified with 0.25 μM each primer, 0.2 mM dNTP, 2.5 mM MgCl_2_, and 1.25 U Taq DNA polymerase (Solis Biodyne, Estonia), in 25 µl volume using the following cycling program: 15 min at 95 °C, then 35 cycles of 30 s at 95 °C, 2 min at 58 °C, 2 min at 72 °C, and final extension 10 min at 72 °C. For the nested PCR, 5 μl of primary PCR products were amplified under the same conditions, except for annealing and extension (1 min). PCR products were detected using 2% agarose gel electrophoresis and ethidium bromide staining. Double strand sequencing of PCR products was performed by Eurofins (Germany). Sequences were analysed with the CLC Main Workbench 20 software. The 3D7 strain of *P. falciparum* (PF3D7_1343700) was used as the reference sequence, to identify polymorphism. All electropherograms were visualized to detect isolates with mixed alleles that were considered to be mutated for the purpose of mutation-frequency estimation. The quality control was assessed by including three blinded quality-control samples in each 96-well sequencing plate.

### Data processing and statistical analyses

The data were compiled in a Microsoft Excel spreadsheet (ver. MS Office 2010). The data were analysed using descriptive statistics (mean, frequency standard deviation and the confidence intervals).

## Results

### Demographic characteristics of the study population

**Fig. 1:**
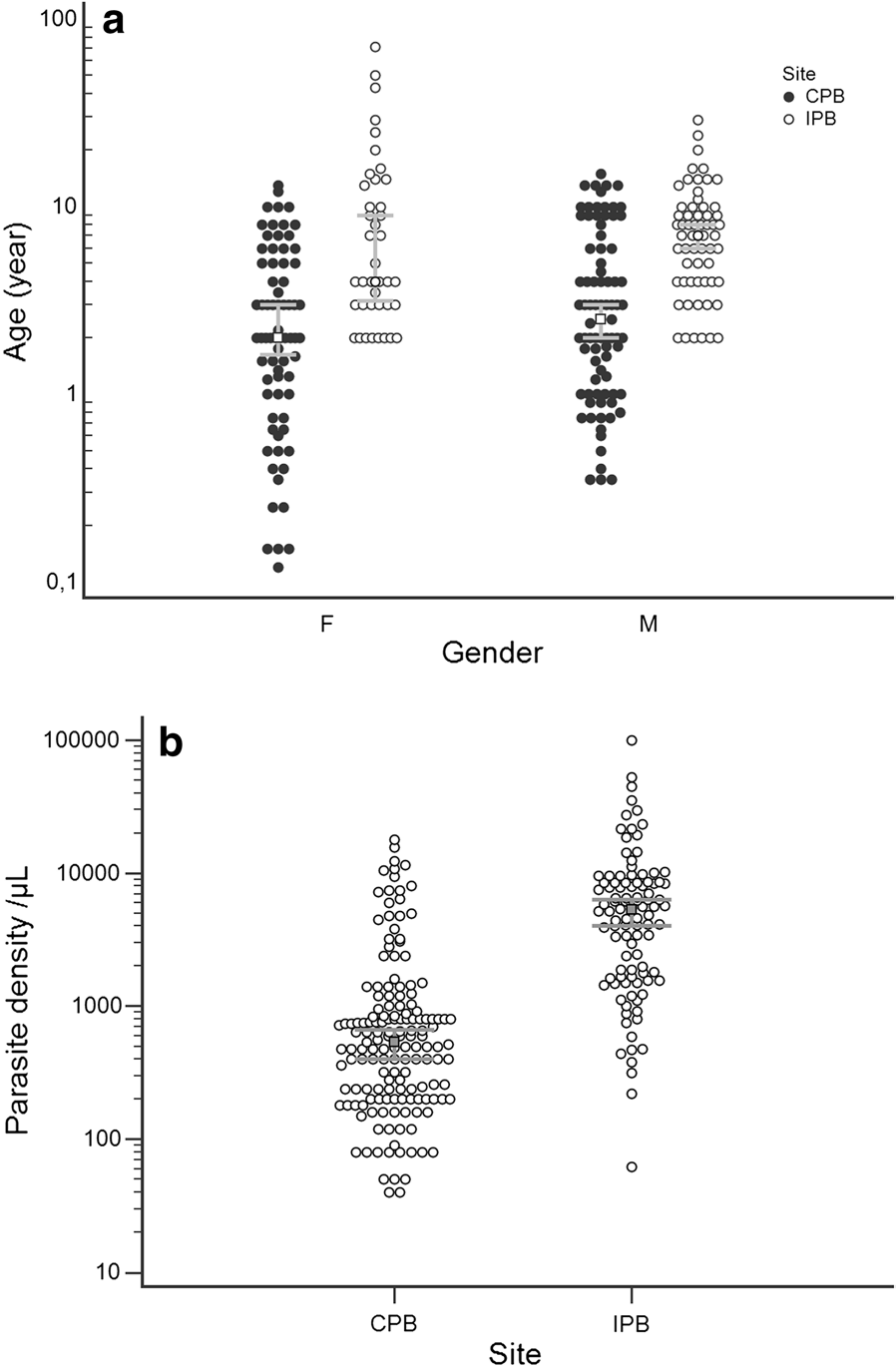
Baseline characteristics of the study population. **a** Distribution of the gender and age according to the site. **b** Distribution of the parasite densities according to the site

The mean age of the patients included in the study was 9.17 years (range: 2 to 71 years) at IPB and 3.75 years (range: 0.12 to 15 years) at the BPC. The difference of the mean age of the populations seeking anti-malarial treatment between the two sites was due that BPC is a pediatric hospital where only patients under 16 years of age are accepted while IPB is opened to the overall population. The sex ratio (M/F) was 1.5 at the IPB and 1.15 at the CPB. The mean parasite density was five times higher in patients recruited at the IPB than those at the CPB (Fig. 1).

### Prevalence of *Plasmodium* species

Of the positive smear samples, 255 could be PCR-amplified using the *Plasmodium* primers: 100% (255/255) were positive for *P. falciparum* and 1.57% (4/255) for *P. ovale*. At the IPB, *P. falciparum* prevalence was 100% (100/100) and *P. ovale* prevalence was 3% (3/100). At the BPC, *P. falciparum* prevalence was 100% (155/155) and *P. ovale* prevalence was 0.65% (1/155). The other two *Plasmodium* species (*Plasmodium malariae* and *Plasmodium vivax*) were not observed.

### Frequency of mutations in the *Pfkelch13* gene

**Fig. 2:**
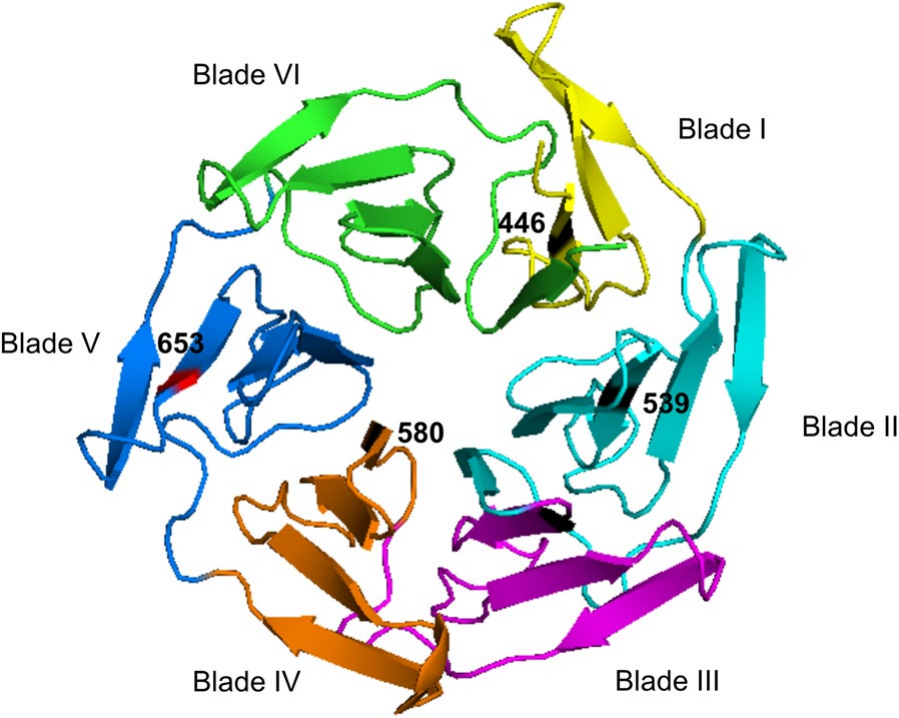
Location of the Y653N mutation in the predicted 3D model of the *Pfkelch13* propeller domain. The predicted structure presents six propeller blades that contain predominantly strands. The locations of the Y653N mutation and the three main Southeast Asian mutations known to confer artemisinin resistance are indicated by spheres

A total of 192 amplicons were sequenced to screen for mutations. Among the 187 interpretable sequences, four mutations (2.14%) were detected, including one non-synonymous mutation (Y653N, 0.54%) and three synonymous mutations (C469C, D464D, A627A, 1.6%) (GenBank Accession Numbers: MT434117-MT434120). The Y653N mutation was observed in a sample collected in 2019 at the CPB (Table 2). The Y653N mutation is located on blade 5 of the *Pfkelch13* propeller domain (Fig. 2). The model predicts that the substitution from aromatic (Y) to polar, non-charged (N) residue at 653 position.

**Table 2:** Polymorphism observed in the *Pfkelch13* in two sites samples collected in Bangui CAR 2017–2019

Sites	Number of samples	SNPs	Codons positions and nitrogenous base	Codons references	Codons mutations	Type of mutations	Frequency (%)
BPC	117	3	D464D C469C Y653N	GA**T** TG**C** **T**AT	GA**C** TG**T** **A**AT	S S NS	2.56
IPB	70	1	A627A	GC**T**	GC**A**	S	1.43
Total	187	4	–	–	–	–	2.14

*S* synonymous; *NS* non-synonymous

## Discussion

The present study provides recent data on the *Plasmodium* species circulating in Bangui, CAR, as well as on the presence of parasites with *Pfkelch13* mutations.

First, the molecular detection of *Plasmodium* DNA in studied isolates showed that both *P. falciparum* and *P. ovale* are circulating in Bangui. Although *P. falciparum* was found in all malaria-positive cases, this species was associated with *P. ovale* in 1.57% of cases. This figure is higher than that of previous observations. A study carried out in 2010 in Bangui estimated a prevalence of 0.3% *P. ovale* [16]. Of note, a co-infection of *P. ovale*, *P. falciparum* and *P. malariae* was observed in Rouen, France in 2017 in an imported malaria case in two children from the CAR [17]. In the other imported malaria study, 4 cases (4/200, i.e. a prevalence of 2%) of *P. ovale* were diagnosed among Peruvian peace-keepers deployed in support of United Nations operations in the CAR from 2016 to 2017 after to return to Peru [18].

Since 2006–2007, resistance to artemisinin has emerged in Southeast Asia, along the Thai–Cambodian border. This resistance, first identified as an increase in parasite clearance times after treatment with artesunate monotherapy or after ACT, is now better understood. It involves early ring-stages that resist treatment by ceasing to grow when exposed to the drug. This phenomenon has been demonstrated by the development of a new in vitro test called the ring-stage survival assay (RSA). Resistant parasites show a proportion of > 1% of parasites that survive after 72 h compared with susceptible parasites (< 1%). Since 2014, these two phenotypes (clinical and in vitro) have been clearly associated with the presence of several non-synonymous mutations in the propeller domain of the *Pfkelch13* gene. The two main hotspots of emergence are located in the Greater Mekong subregion in Southeast Asia, where the parasites carrying the C580Y or the F446I mutations are now dominant [12]. Other single point mutations (N458Y, Y493H, R539T, I543T, M476I, P553L and R561H) have also been validated as conferring resistance to artemisinin [19]. In addition, some mutations (P441L, G449A, C469F, P527H, N537I, G538V, V568G, P574L, F673I and A675V) are candidates suspected to be associated with artemisinin resistance [19]. In sub-Saharan Africa, validated or candidate mutations associated with resistance (R539T, P574L) have been observed in Angola, Equatorial Guinea and in Rwanda, whereas mutations potentially associated with artemisinin resistance (M476I) have been detected in Senegal [20–23]. A particular case was the observation of a local mutation (M579I) in Equatorial Guinea [24]. This mutation was shown to be associated with artemisinin resistance, while the A578S mutation, common in Africa, was not found to be associated with artemisinin resistance [25]. Here, analyses of samples collected in Bangui between 2017 and 2019 demonstrate the presence of one non-synonymous mutation (Y653N) and three synonymous mutations (frequency of 2.1%). This frequency of *Pfkelch13* mutants is similar to those observed in neighbouring countries (Brazzaville Congo, certain regions of Cameroon), which showed frequencies of 1.57% and 2.9%, respectively [26, 27], confirming the absence of artemisinin resistance in Central Africa [20]. The only previous data on *Pfkelch13* polymorphism performed in 2014 in the CAR (K13 artemisinin resistance multicenter resistance, KARMA study), revealed a 4.5% prevalence of non-synonymous mutations [12]. The difference or the fluctuation in the frequency of non-synonymous mutations in the CAR in 2014 and that of Bangui in 2017–2019 is likely due to the representativeness of the samples tested, but also to the fact that mutants appear at random and disappear probably because they do not have a selective advantage compared with wild strains [28]. The previously validated or candidate resistance-associated mutations were not detected in our study. Interestingly, the A578S codon, common in Africa, was not observed either. The sole non-synonymous mutation detected here has never been reported in other African countries. It remains to be seen if this mutation is associated with artemisinin resistance. It is possible that local mutant strains resistant to artemisinin can emerge in addition to the risk of spread of the resistant Southeast Asian strain, as observed previously with strains resistant to chloroquine or sulfadoxine–pyrimethamine.

The dynamics of resistance and its emergence are likely to be complex, particularly due to the interactions specific to each world (sub) region [29]. Similar studies in Cameroon and Nigeria have shown large differences according to region and time period, which makes it difficult to determine the spatio-temporal dynamics on polymorphism [6, 30]. Some countries in this African subregion (Congo, Gabon, and DRC) have revealed the presence of parasites polymorphic for the A578S allele, others have detected novel alleles and still others, particularly in Benin, have not detected any polymorphism [31, 32].

If the Y653N mutation increases in frequency in subsequent studies, it will then be necessary to assess its in vivo impact on the parasite clearance half-life in patients treated with ACT and its susceptibility in vitro to confirm or disprove its association with artemisinin resistance.

## Conclusion

This study demonstrates the presence of a strain carrying a non-synonymous mutation in Bangui. Neither non-synonymous mutations involved in previously demonstrated in vitro and in vivo resistance nor the candidate resistance mutations were detected. The A578S mutant, although not associated with resistance but frequently found in Africa, was not observed among the tested samples. The novel mutation detected here is yet another mutation to add to the limited list of non-synonymous mutations detected in Africa. Additional studies with samples collected throughout the CAR are needed to confirm this narrow polymorphism profile.

## Data Availability

The database of this study is available from the corresponding author upon request.

## Abbreviations

ACT: Artemisinin-based combination therapy
CAR: Central African Republic
IPB: Institut Pasteur in Bangui
WHO: World Health Organization
PCB: Paediatric Complex in Bangui
*Pfkelch13*: *Plasmodium falciparum kelch 13* gene
RSA: Ring-stage Survival Assay
